# Supplementary material for: Differential Expression of Nicotine Acetylcholine Receptors Associates with Human Breast Cancer and Mediates Antitumor Activity of αO-Conotoxin GeXIVA
Source: Mar Drugs. 2020 Jan 17;18(1):61. doi: 10.3390/md18010061 (PMC7024346; doi:10.3390/md18010061)
Supplement: Supplementary file 1 [file marinedrugs-18-00061-s001.zip › Figure S1 reverse-transcription PCR.docx]

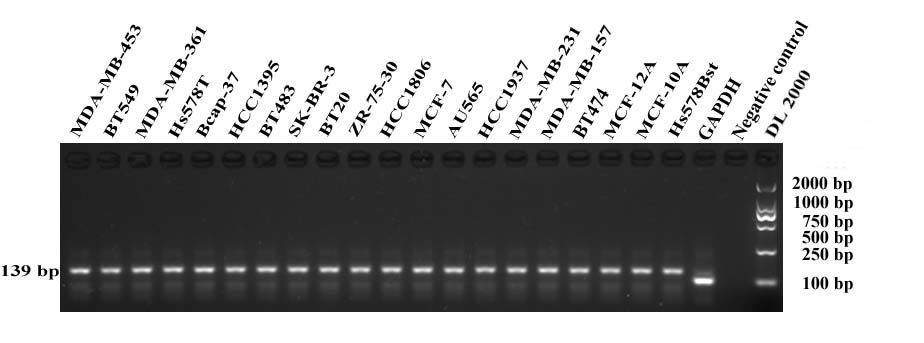


**Figure S1A. Reverse transcription PCR (RT-PCR) ampliﬁcation of** **α3 nAChR sequences from human breast cells**

Agarose gel electrophoresis of PCR products ampliﬁed from RNA samples reverse-transcribed to cDNA. 1μg of puriﬁed total RNA extracted from human breast cells cultured were reverse-transcribed to cDNA and ampliﬁed for 30 cycles with one pairs of α3 nAChR subtype-speciﬁc primers. The results confirmed that the products ampliﬁed from normal human breast cell lines (MCF-10A, MCF-12A and Hs578Bst) and human breast cancer cell lines (MDA-MB-157, MDA-MB-231, MDA-MB-361, MDA-MB-453, BT20, BT474, BT483, BT549, HCC1395, HCC1806, HCC1937, AU565, SK-BR-3, MCF-7, Bcap-37, ZR-75-30, Hs578T)cDNA contained authentic LBD fragments sequences of human a3 nAChR subtypes. The bands were consistent with the expected sizes, 139 bp and 103 bp for α3 nAChR and GAPDH. The size of each ampliﬁcation product was determined using the DL2000 DNA Marker loaded.


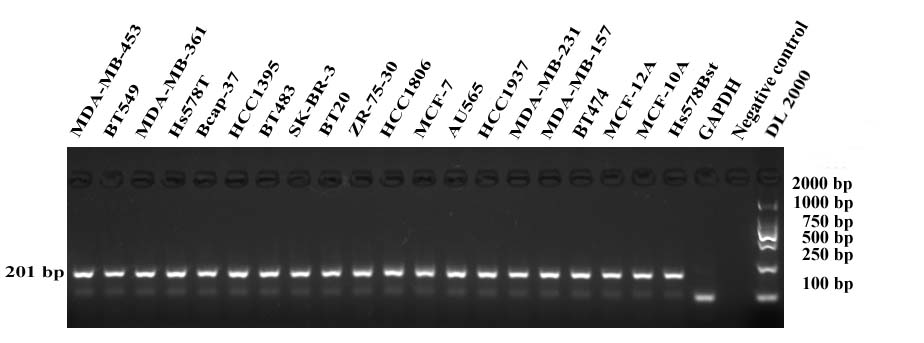


**Figure S1B. Reverse transcription PCR (RT-PCR) ampliﬁcation of α4 nAChR sequences from human breast cells**

Agarose gel electrophoresis of PCR products ampliﬁed from RNA samples reverse-transcribed to cDNA. 1μg of puriﬁed total RNA extracted from human breast cells cultured were reverse-transcribed to cDNA and ampliﬁed for 30 cycles with one pairs of α4 nAChR subtype-speciﬁc primers. The results confirmed that the products ampliﬁedfrom normal human breast cell lines (MCF-10A, MCF-12A and Hs578Bst) and human breast cancer cell lines (MDA-MB-157, MDA-MB-231, MDA-MB-361, MDA-MB-453, BT20, BT474, BT483, BT549, HCC1395, HCC1806, HCC1937, AU565, SK-BR-3, MCF-7, Bcap-37, ZR-75-30, Hs578T)cDNA contained authentic LBD fragments sequences of human a4 nAChR subtypes. The bands were consistent with the expected sizes, 201 bp and 103 bp for α4 nAChR and GAPDH. The size of each ampliﬁcation product was determined using the DL2000DNA Marker loaded.


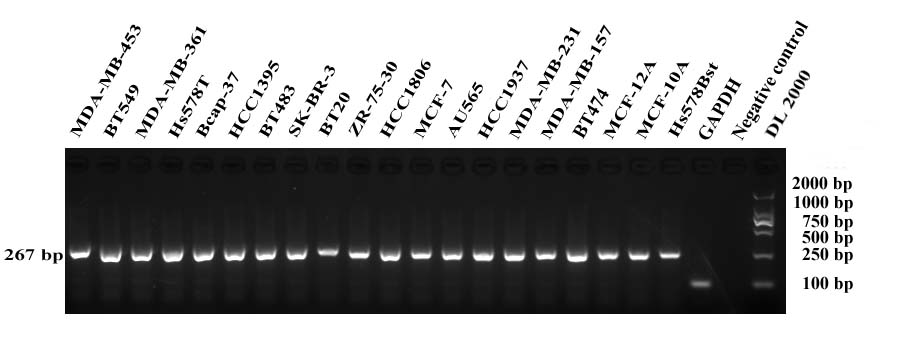


**Figure S1C. Reverse transcription PCR (RT-PCR) ampliﬁcation of α5 nAChR sequences from human breast cells**

Agarose gel electrophoresis of PCR products ampliﬁed from RNA samples reverse-transcribed to cDNA. 1μg of puriﬁed total RNA extracted from human breast cells cultured were reverse-transcribed to cDNA and ampliﬁed for 30 cycles with onepairs of α5 nAChR subtype-speciﬁc primers. The results confirmed that the products ampliﬁed from normal human breast cell lines (MCF-10A, MCF-12A and Hs578Bst) and human breast cancer cell lines (MDA-MB-157, MDA-MB-231, MDA-MB-361, MDA-MB-453, BT20, BT474, BT483, BT549, HCC1395, HCC1806, HCC1937, AU565, SK-BR-3, MCF-7, Bcap-37, ZR-75-30, Hs578T)cDNA contained authentic LBD fragments sequences of human a5 nAChR subtypes. The bands were consistent with the expected sizes, 267 bp and 103 bp for α5nAChRand GAPDH. The size of each ampliﬁcation product was determined using the DL2000 DNA Marker loaded.


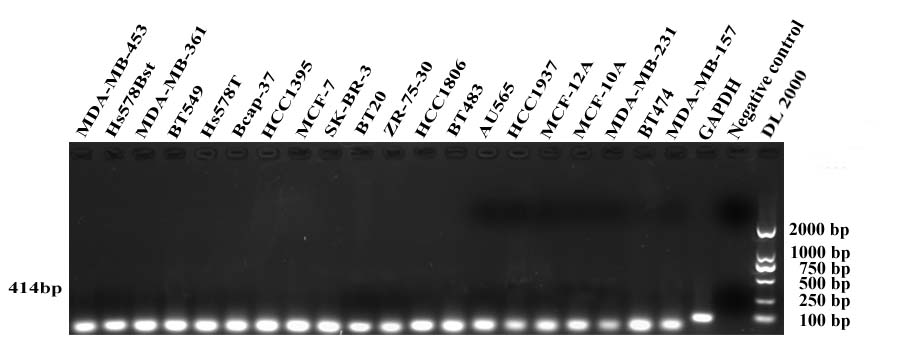


**Figure S1D. Reverse transcription PCR (RT-PCR) ampliﬁcation of α6 nAChR sequences from human breast cells**

Agarose gel electrophoresis of PCR products ampliﬁed from RNA samples reverse-transcribed to cDNA. 1μg of puriﬁed total RNA extracted from human breast cells cultured were reverse-transcribed to cDNA and ampliﬁed for 30 cycles with one pairs of α6 nAChR subtype-speciﬁc primers. The results confirmed that 20 cell lines didn't produce a specific amplification fragment for α6 nAChR subunit’s LBD cDNA with the expected size fragments. The bands were consistent with the expected sizes, 414 bp and 103 bp for α6 nAChRand GAPDH. The size of each ampliﬁcation product was determined using the DL2000 DNA Marker loaded.


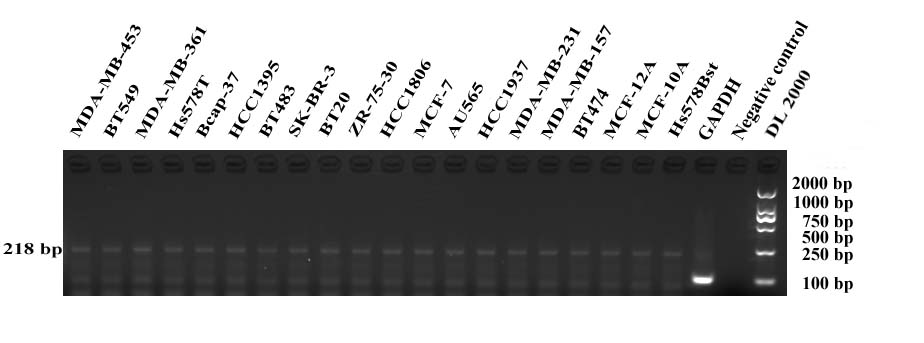


**Figure S1E. Reverse transcription PCR (RT-PCR) ampliﬁcation of α7 nAChR sequences from human breast cells**

Agarose gel electrophoresis of PCR products ampliﬁed from RNA sample sreverse-transcribed to cDNA. 1μg of puriﬁed total RNA extracted from human breast cells cultured were reverse-transcribed tocDNA and ampliﬁed for 30 cycles with one pairs of α7 nAChR subtype-speciﬁc primers. The results confirmed that the products ampliﬁedfrom normal human breast cell lines (MCF-10A, MCF-12A and Hs578Bst) and human breast cancer cell lines (MDA-MB-157, MDA-MB-231, MDA-MB-361, MDA-MB-453, BT20, BT474, BT483, BT549, HCC1395, HCC1806, HCC1937, AU565, SK-BR-3, MCF-7, Bcap-37, ZR-75-30, Hs578T) cDNA contained authentic LBD fragments sequences of human a7 nAChR subtypes. The bands were consistent with the expected sizes, 218 bp and 103 bp for α7nAChRand GAPDH. The size of each ampliﬁcation product was determined using the DL2000 DNA Marker loaded.


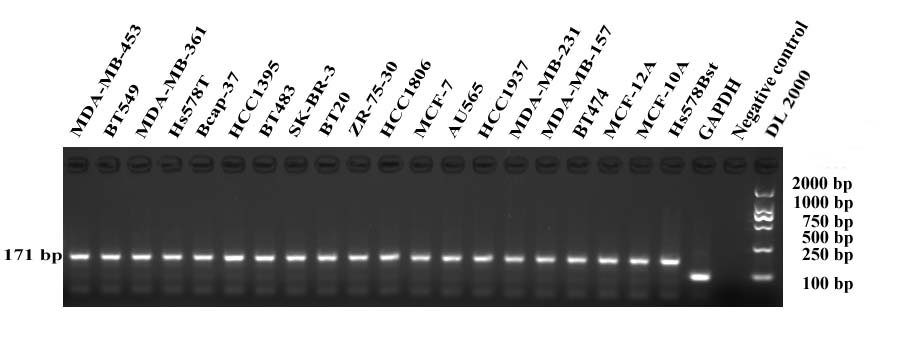


**Figure S1F. Reverse transcription PCR (RT-PCR) ampliﬁcation of α9 nAChR sequences from human breast cells**

Agarose gel electrophoresis of PCR products ampliﬁed from RNA samples reverse-transcribed to cDNA. 1μg of puriﬁed total RNA extracted from human breast cells cultured were reverse-transcribed to cDNA and ampliﬁed for 30 cycles with one pairs of α9 nAChR subtype-speciﬁc primers. The results confirmed that the products ampliﬁedfrom normal human breast cell lines (MCF-10A, MCF-12A and Hs578Bst) and human breast cancer cell lines (MDA-MB-157, MDA-MB-231, MDA-MB-361, MDA-MB-453, BT20, BT474, BT483, BT549, HCC1395, HCC1806, HCC1937, AU565, SK-BR-3, MCF-7, Bcap-37, ZR-75-30, Hs578T)cDNA contained authentic LBD fragments sequences of human a9 nAChR subtypes. The bands were consistent with the expected sizes, 171 bp and 103 bp for α9 nAChR and GAPDH. The size of each ampliﬁcation product was determined using the DL2000 DNA Marker loaded.


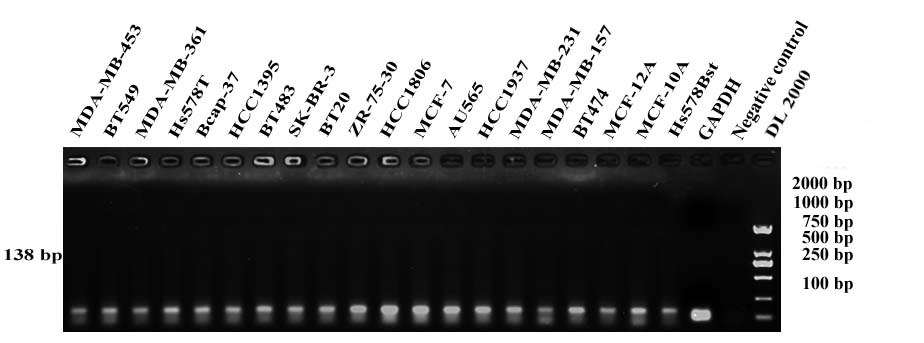


**Figure S1G. Reverse transcription PCR (RT-PCR) ampliﬁcation of α10 nAChR sequences from human breast cells**

Agarose gel electrophoresisofPCR products ampliﬁed from RNA samples reverse-transcribed to cDNA. 1μg of puriﬁed total RNA extracted from human breast cells cultured were reverse-transcribed to cDNA and ampliﬁed for 30 cycles with one pairs of α10 nAChR subtype-speciﬁc primers. The results confirmed that the products ampliﬁedfrom normal human breast cell lines (MCF-10A, MCF-12A and Hs578Bst) and human breast cancer cell lines (MDA-MB-157, MDA-MB-231, MDA-MB-361, MDA-MB-453, BT20, BT474, BT483, BT549, HCC1395, HCC1806, HCC1937, AU565, SK-BR-3, MCF-7, Bcap-37, ZR-75-30, Hs578T)cDNA contained authentic LBD fragments sequences of human a10 nAChR subtypes. The bands were consistent with the expected sizes, 138 bp and 103 bp for α10nAChRand GAPDH. The size of each ampliﬁcation product was determined using the DL2000 DNA Marker loaded.


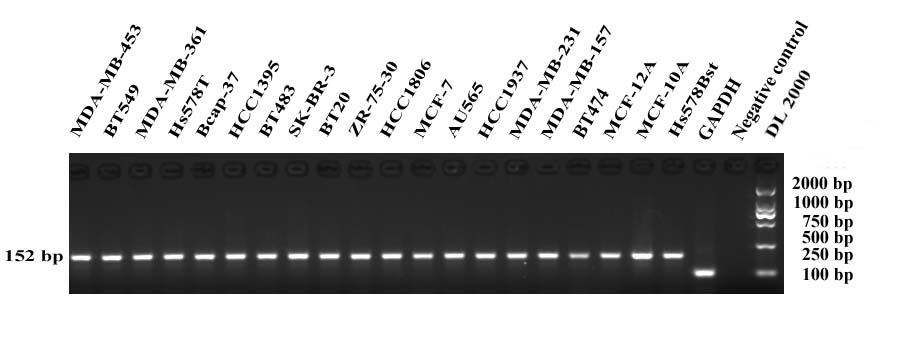


**Figure S1H. Reverse transcription PCR (RT-PCR) ampliﬁcation of β2 nAChR sequences from human breast cells**

Agarose gel electrophoresis of PCR products ampliﬁed from RNA samples reverse-transcribed to cDNA. 1μg of puriﬁed total RNA extracted from human breast cells cultured were reverse-transcribed to cDNA and ampliﬁed for 30 cycles with one pairs of β2nAChR subtype-speciﬁc primers. The results confirmed that the products ampliﬁedfrom normal human breast cell lines (MCF-10A, MCF-12A and Hs578Bst) and human breast cancer cell lines (MDA-MB-157, MDA-MB-231, MDA-MB-361, MDA-MB-453, BT20, BT474, BT483, BT549, HCC1395, HCC1806, HCC1937, AU565, SK-BR-3, MCF-7, Bcap-37, ZR-75-30, Hs578T)cDNA contained authentic LBD fragments sequences of human β2 nAChR subtypes. The bands were consistent with the expected sizes, 152 bp and 103 bp for β2nAChRand GAPDH. The size of each ampliﬁcation product was determined using the DL2000 DNA Marker loaded.


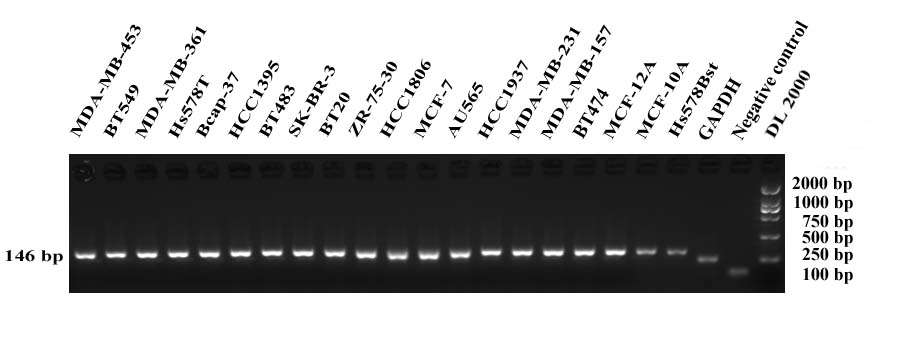


**Figure S1I. Reverse transcription PCR (RT-PCR) ampliﬁcation of β3 nAChR sequences from human breast cells**

Agarose gel electrophoresis of PCR products ampliﬁed from RNA samples reverse-transcribed to cDNA. 1μg of puriﬁed total RNA extracted from human breast cells cultured were reverse-transcribed to cDNA and ampliﬁed for 30 cycles with one pairs of β3 nAChR subtype-speciﬁc primers. The results confirmed that the products ampliﬁedfrom normal human breast cell lines (MCF-10A, MCF-12A and Hs578Bst) and human breast cancer cell lines (MDA-MB-157, MDA-MB-231, MDA-MB-361, MDA-MB-453, BT20, BT474, BT483, BT549, HCC1395, HCC1806, HCC1937, AU565, SK-BR-3, MCF-7, Bcap-37, ZR-75-30, Hs578T)cDNA contained authentic LBD fragments sequences of human β3 nAChR subtypes. The bands were consistent with the expected sizes, 146 bp and 103 bp for β3nAChRand GAPDH. The size of each ampliﬁcation product was determined using the DL2000DNA Marker loaded.


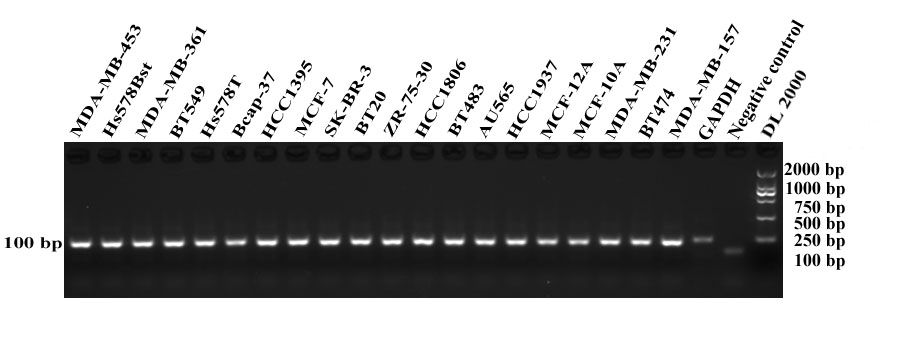


**Figure S1J. Reverse transcription PCR (RT-PCR) ampliﬁcation of β4 nAChR sequences from human breast cells**

Agarose gel electrophoresis of PCR products ampliﬁed from RNA samples reverse-transcribed to cDNA. 1μg of puriﬁed total RNA extracted from human breast cells cultured were reverse-transcribed tocDNA and ampliﬁed for 30 cycles with one pairs of β4 nAChR subtype-speciﬁc primers. The results confirmed that the products ampliﬁedfrom normal human breast cell lines (MCF-10A, MCF-12A and Hs578Bst) and human breast cancer cell lines (MDA-MB-157, MDA-MB-231, MDA-MB-361, MDA-MB-453, BT20, BT474, BT483, BT549, HCC1395, HCC1806, HCC1937, AU565, SK-BR-3, MCF-7, Bcap-37, ZR-75-30, Hs578T) cDNA contained authentic LBD fragments sequences of human β4 nAChR subtypes. The bands were consistent with the expected sizes, 100 bp and 103 bp for β4 nAChRand GAPDH. The size of each ampliﬁcation product was determined using the DL2000 DNA Marker loaded.
