# Supplementary material for: Differential Expression of Nicotine Acetylcholine Receptors Associates with Human Breast Cancer and Mediates Antitumor Activity of αO-Conotoxin GeXIVA
Source: Mar Drugs. 2020 Jan 17;18(1):61. doi: 10.3390/md18010061 (PMC7024346; doi:10.3390/md18010061)
Supplement: Supplementary file 1 [file marinedrugs-18-00061-s001.zip › Figure S3 protein expression by FCM.docx]

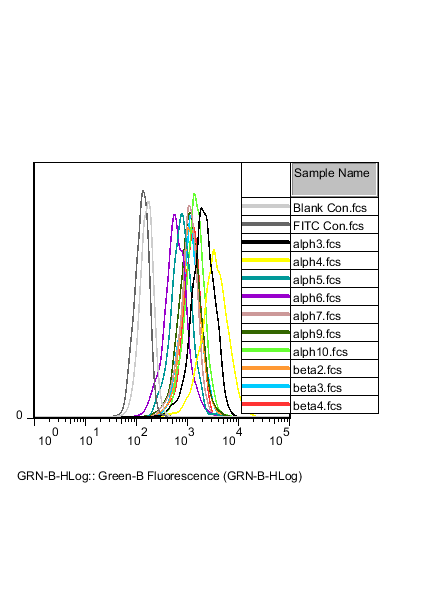


**B. HCC937**


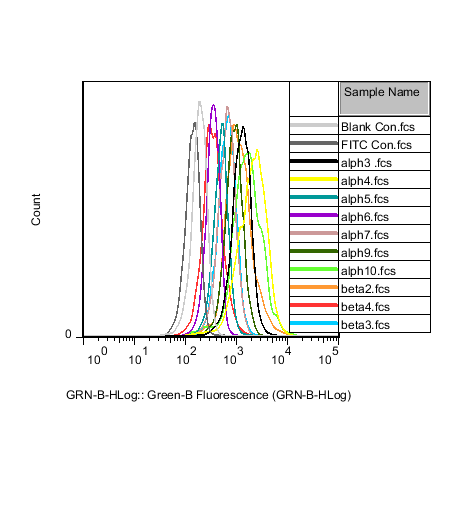

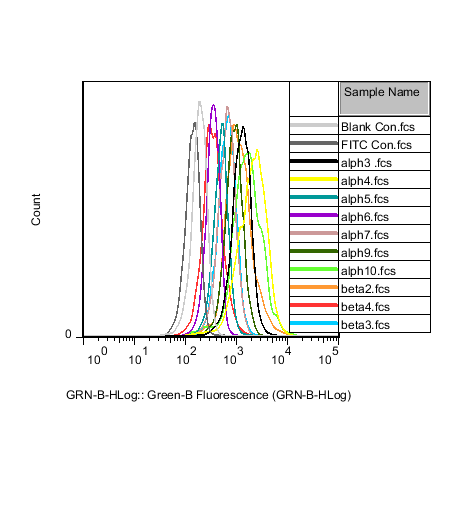


**A. HCC1395**


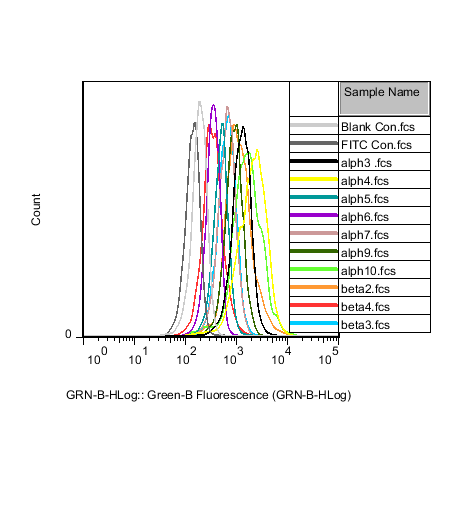

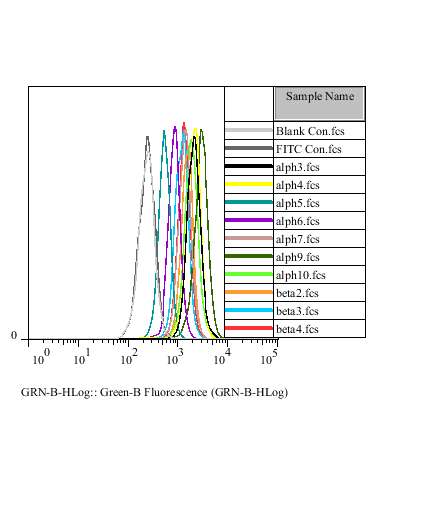


**D. BT549**


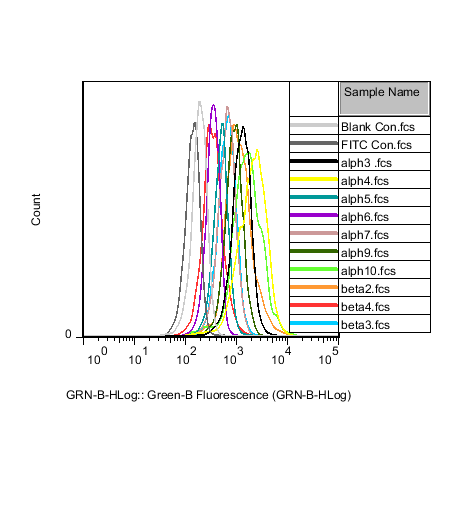

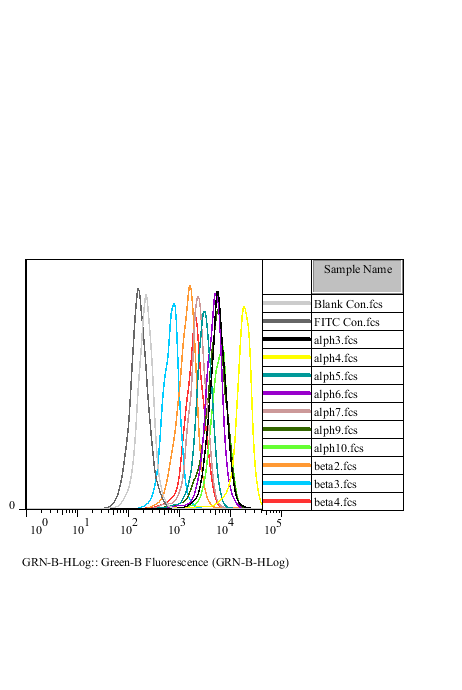


**C. HCC1806**


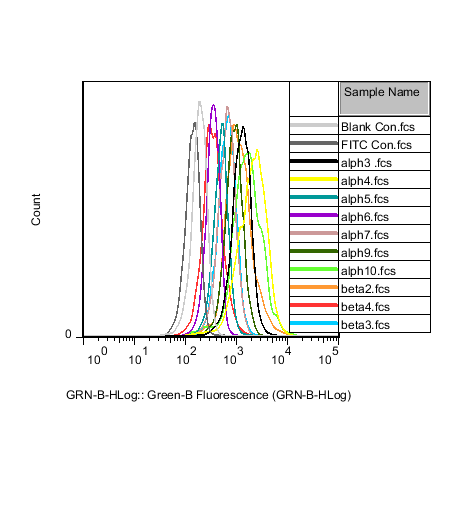


**
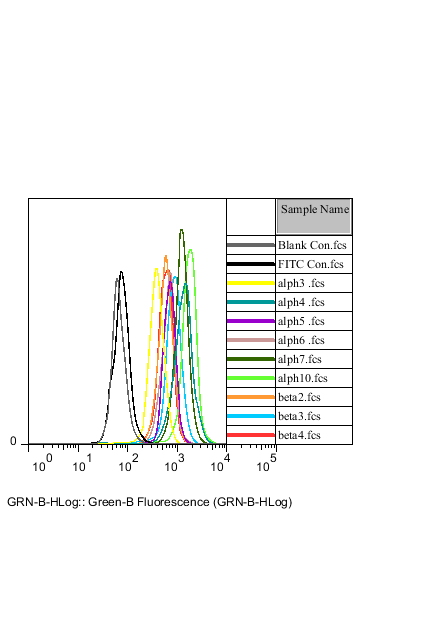
**


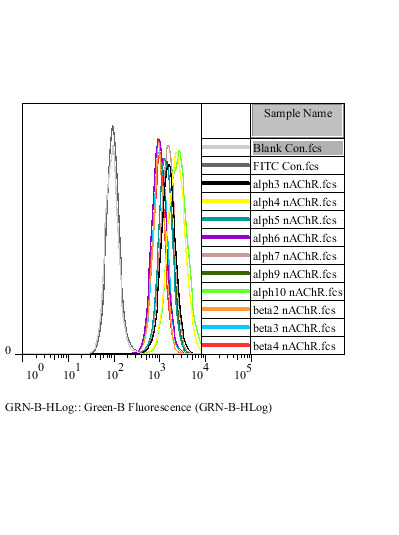


**E. BT483**


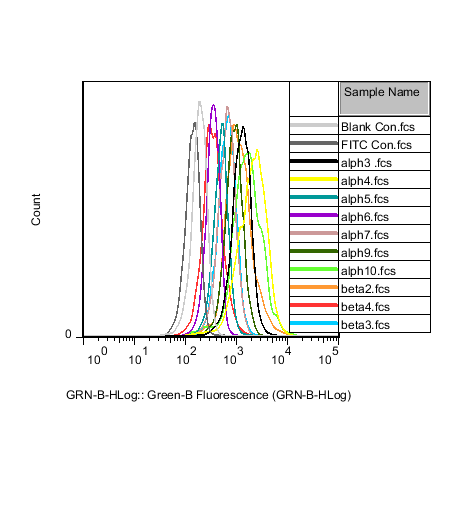


**
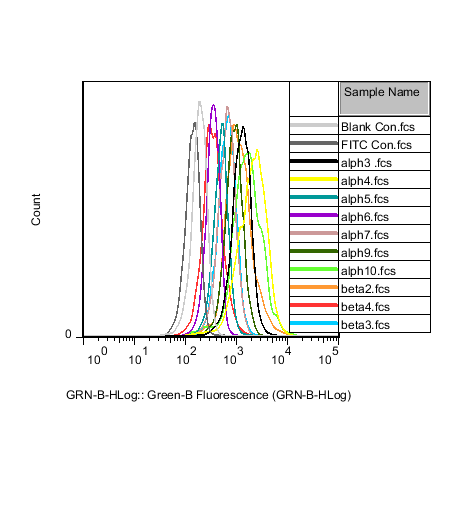
**

**F. BT20**


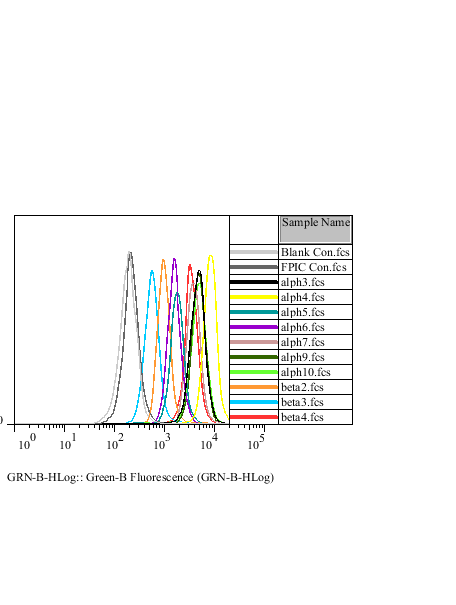


**H. MDA-MB-231**


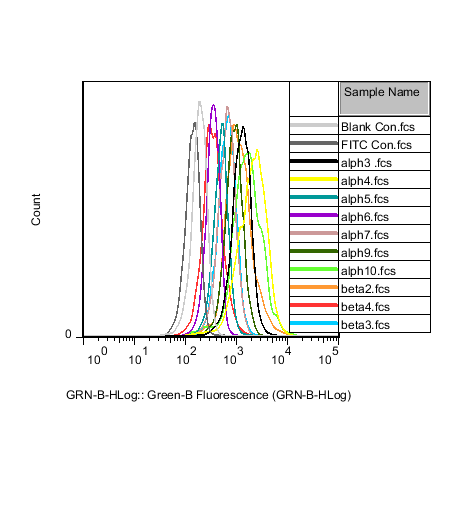

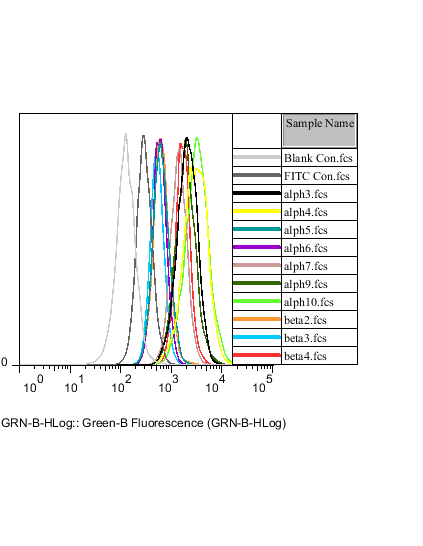


**G. MDA-MB-453**


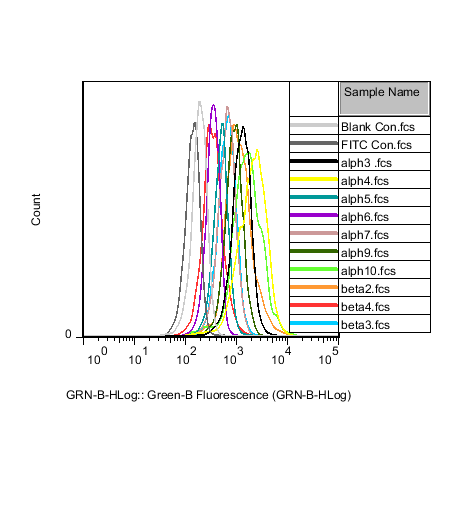

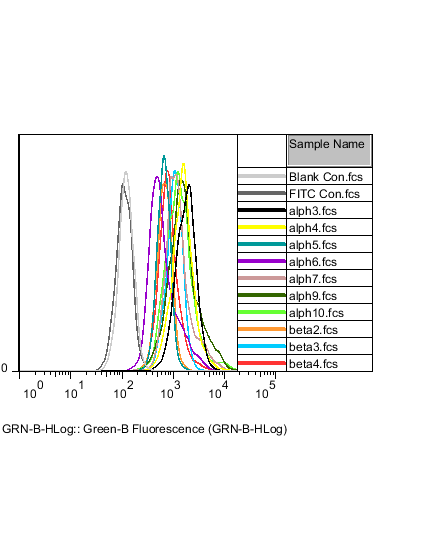


**J. Bcap-37**


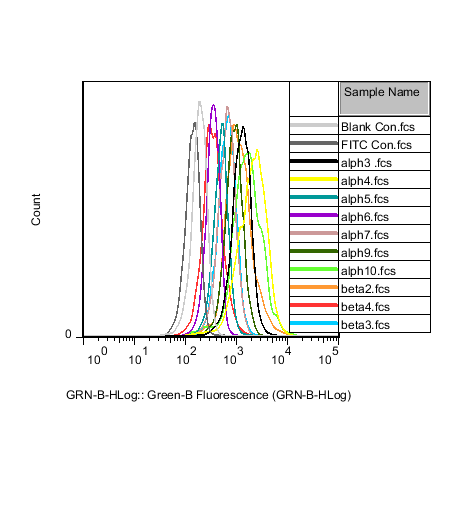

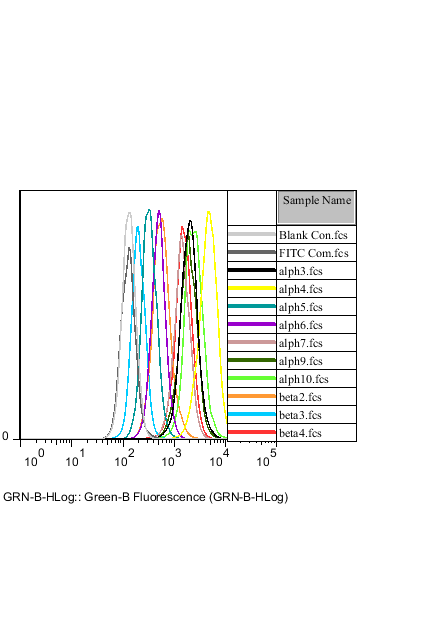


**I. MDA-MB-361**


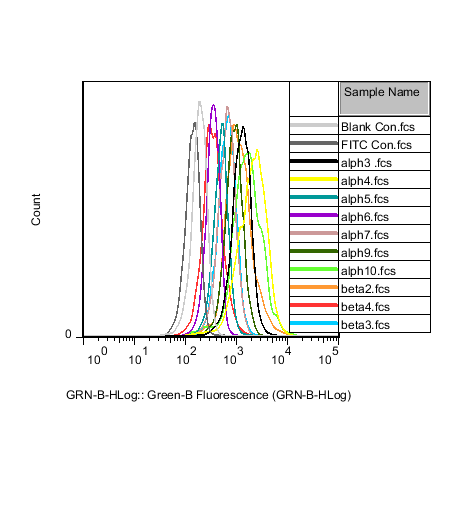

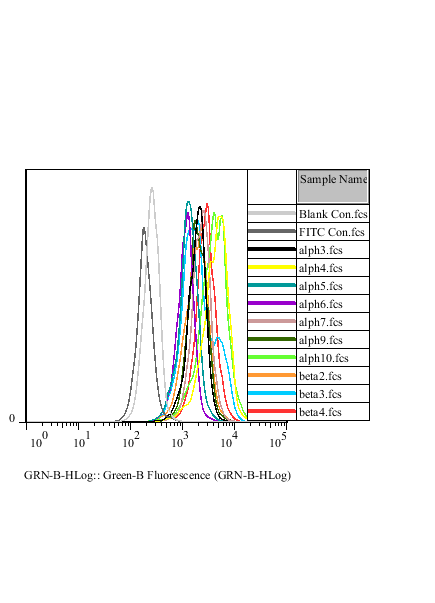


**L.Hs578T**


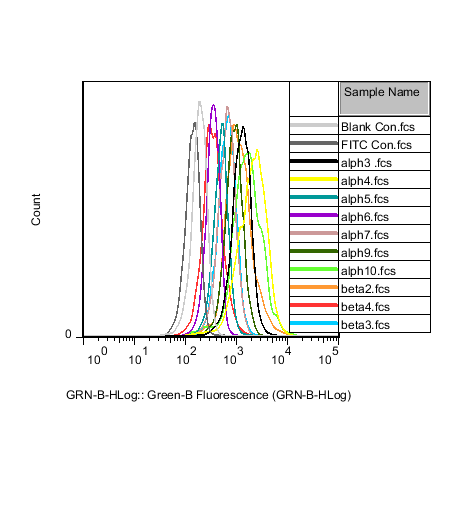

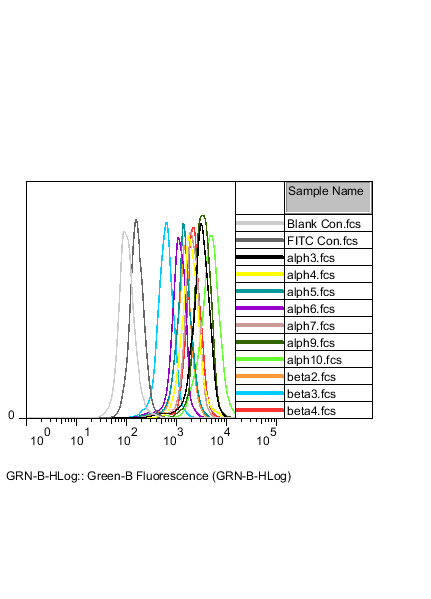


**K. ZR-75-30**


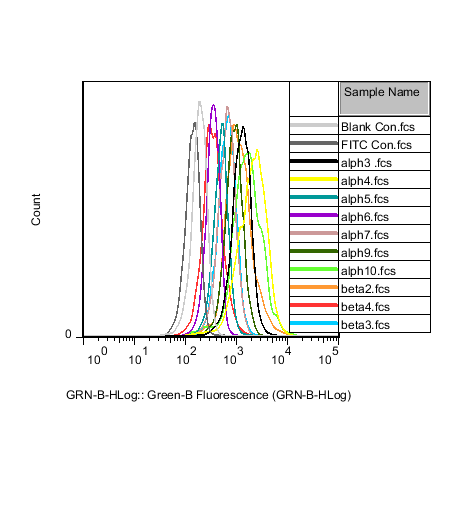

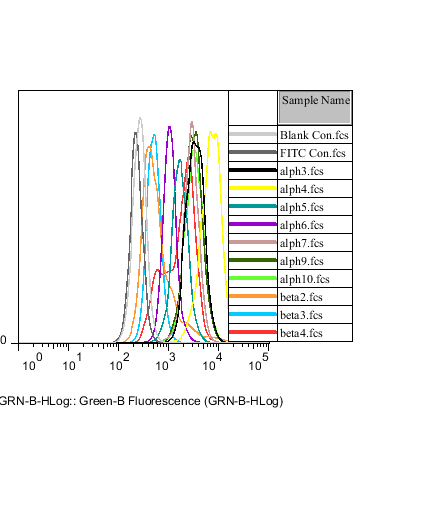


**N. AU565**


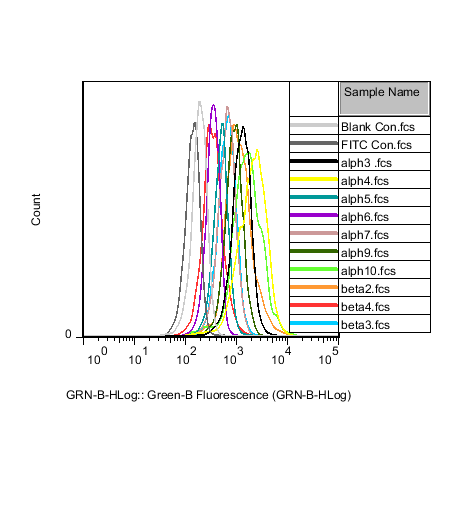

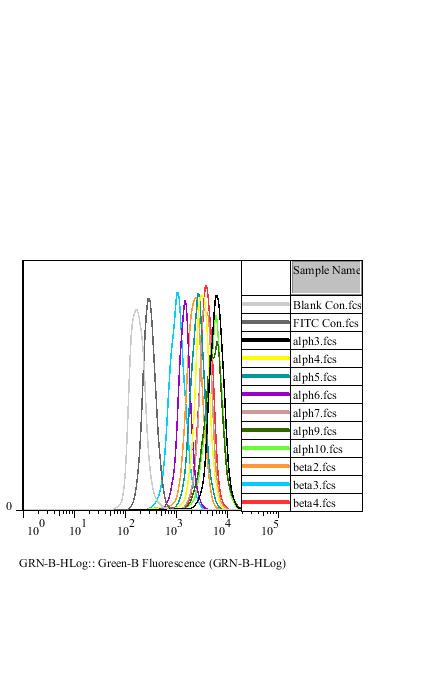


**M. BT474**


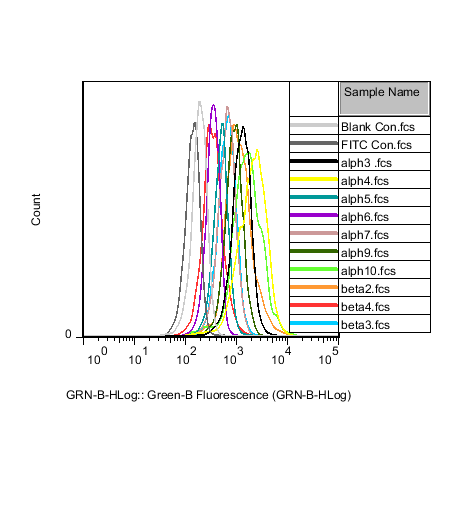

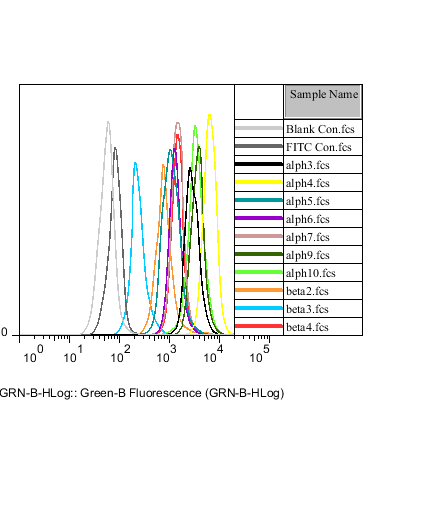


**P. MCF-7**


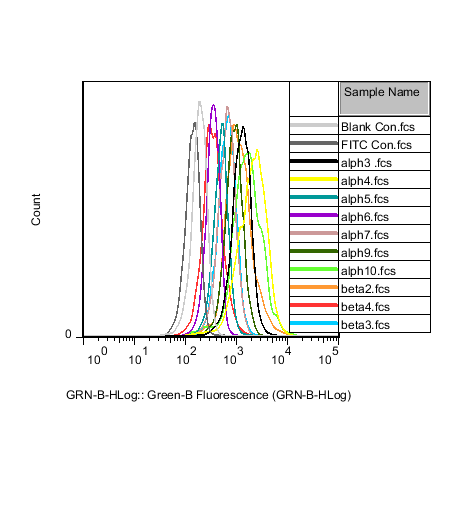

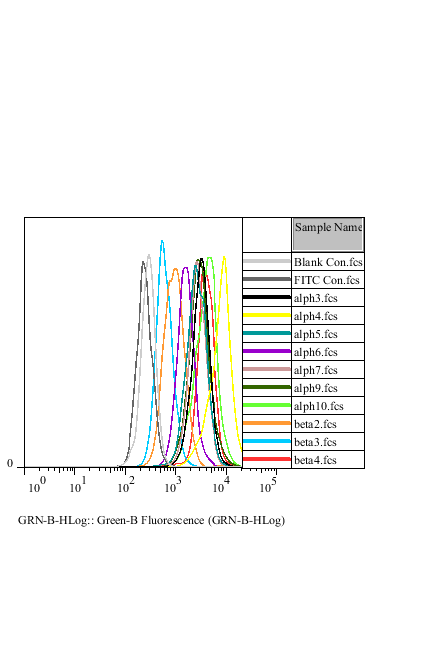


**O. SK-BR-3**


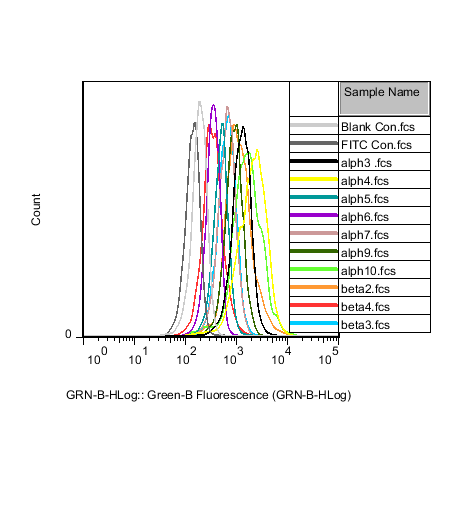

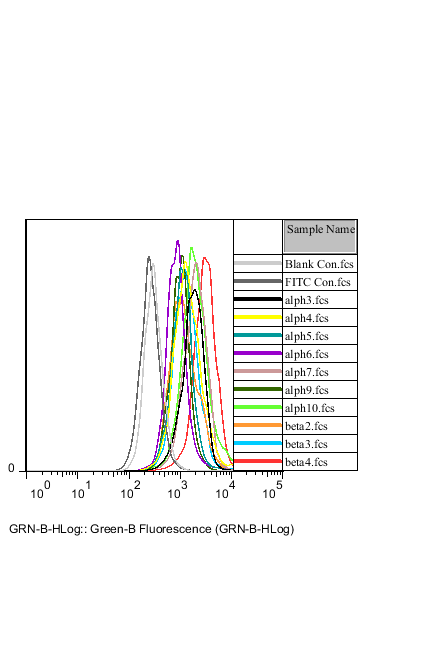


**Q. Hs578BST**


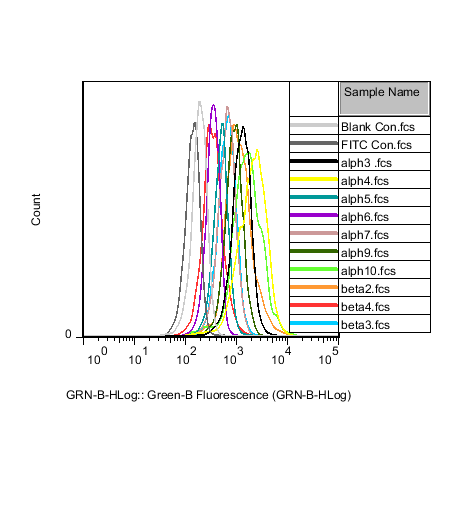


**Figure S3 (A-Q). Flow cytometry analysis of nAChRs staining intensity under antibody treatment.**

Histograms of cell distribution according to green fluorescence intensity for cell incubated with anti-nAChRs antibodies and FITC-conjugated Goat anti-rabbit Ig(G+L) are shown.
