# Supplementary material for: Differential Expression of Nicotine Acetylcholine Receptors Associates with Human Breast Cancer and Mediates Antitumor Activity of αO-Conotoxin GeXIVA
Source: Mar Drugs. 2020 Jan 17;18(1):61. doi: 10.3390/md18010061 (PMC7024346; doi:10.3390/md18010061)
Supplement: Supplementary file 1 [file marinedrugs-18-00061-s001.zip › Figure S4 the difference analysis of protein.docx]

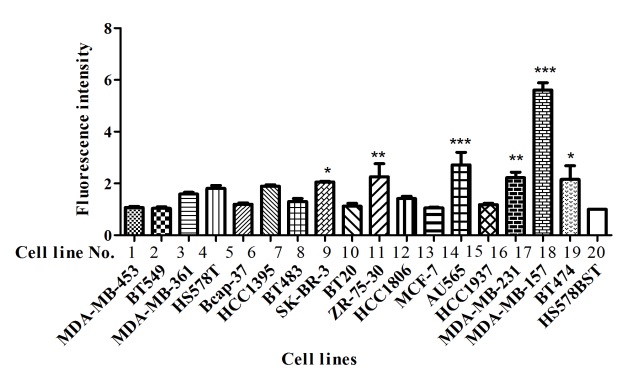


**J. β4 nAChR**


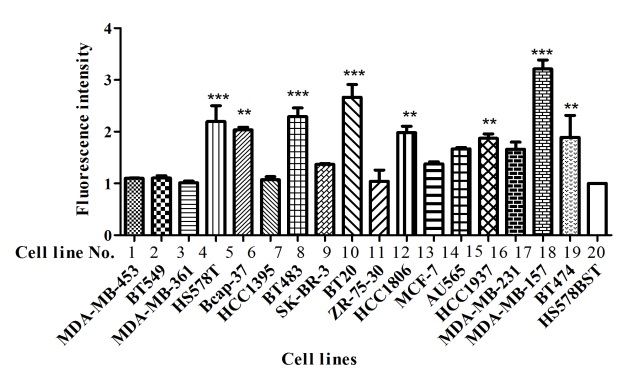


**I. β3 nAChR**


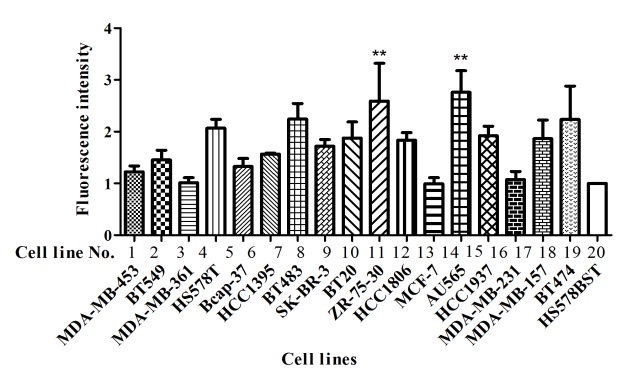


**H. β2 nAChR**


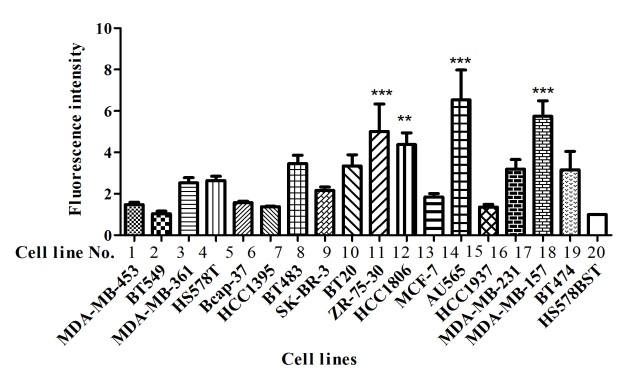


**G. α10 nAChR**


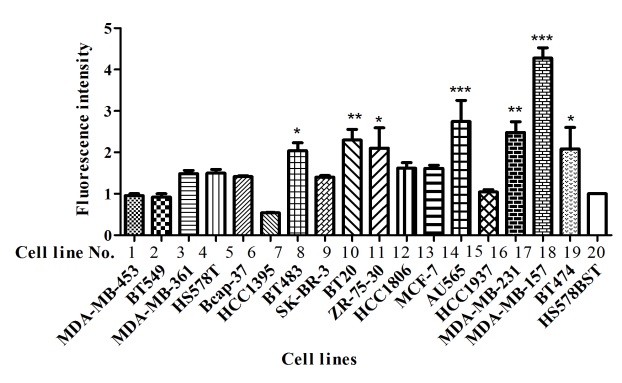


**E. α7 nAChR**


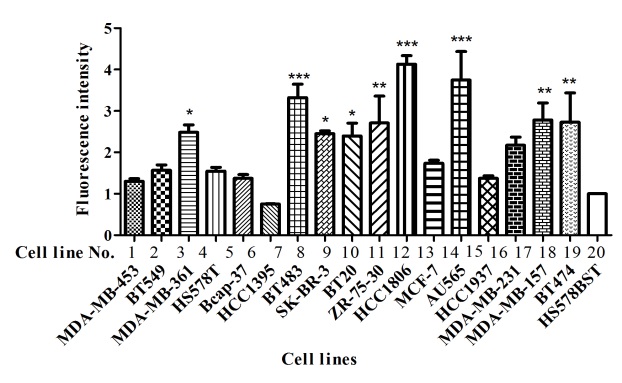


**C. α5 nAChR**


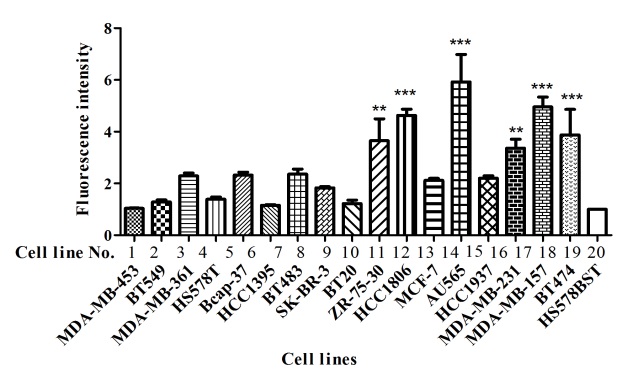


**A. α3 nAChR**


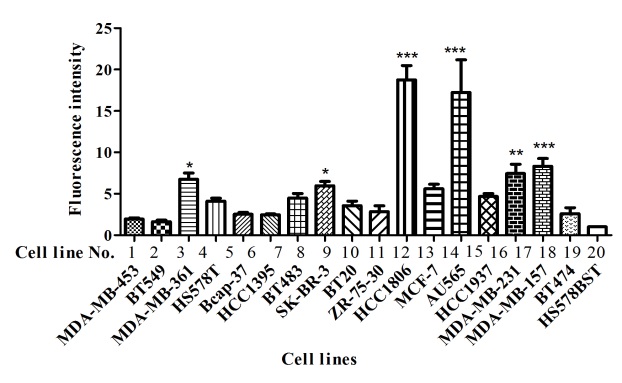


**B. α4 nAChR**


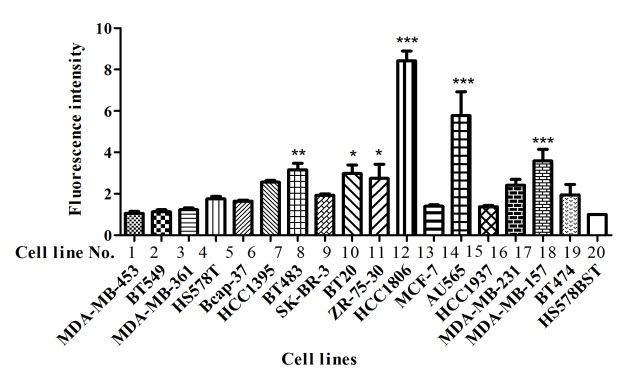


**D. α6 nAChR**

**Figure S4 (A-J). Flow cytometry analysis of nAChRs staining intensity under antibody treatment.**

Mean staining intensities for nAChRs in breast cancer cells treated with antibody compared with the normal cells. Data are expressed as mean ± SEM, n = 3. *p^***^*﹤0.001, breast cancer cells vs. normal cells (HS578BST), ANOVA with Dunnett’s multiple comparisons post hoc test.
