# Supplementary figures and images for: Differential Expression of Nicotine Acetylcholine Receptors Associates with Human Breast Cancer and Mediates Antitumor Activity of αO-Conotoxin GeXIVA
Source: Mar Drugs. 2020 Jan 17;18(1):61. doi: 10.3390/md18010061 (PMC7024346; doi:10.3390/md18010061)

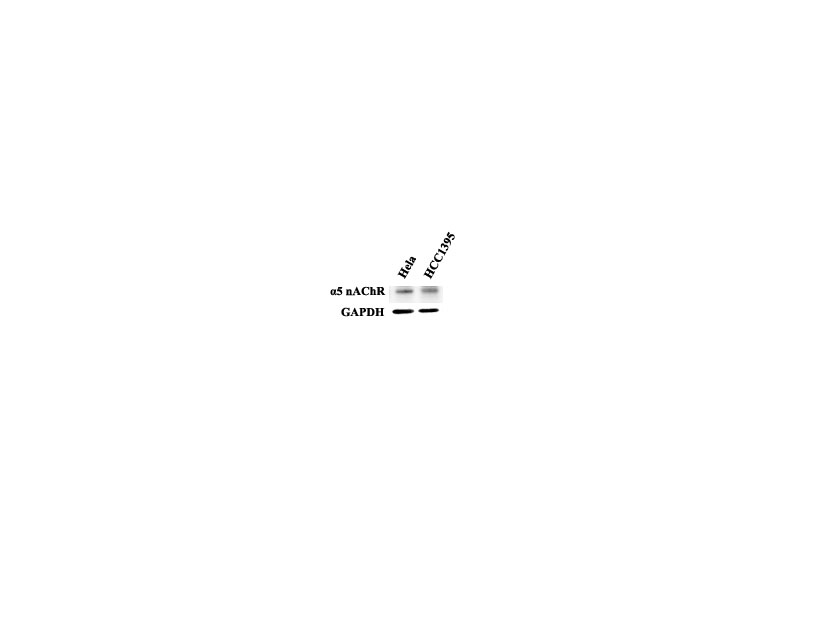

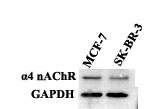


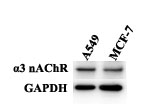


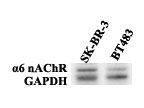


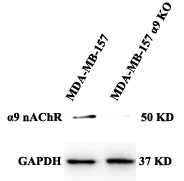


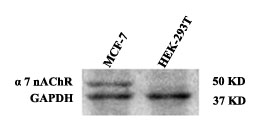


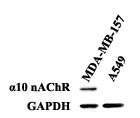


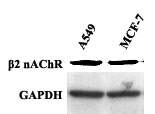


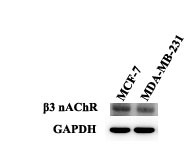


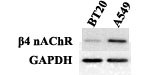


**Figure S2. The protein expression of nAChR in different cell lines by Western blotting.**

Supplement: Supplementary file 1 [file marinedrugs-18-00061-s001.zip › Figure S2 The specificity of antibodies.docx]
